# Leukocyte and Platelet-Rich Plasma (L-PRP) in Tendon Models: A Systematic Review and Meta-Analysis of *in vivo*/*in vitro* Studies

**DOI:** 10.1155/2022/5289145

**Published:** 2022-12-15

**Authors:** Xueli Liu, Yulin Li, Li Shen, Maorun Yan

**Affiliations:** ^1^Department of Rehabilitation, Sichuan Vocational College of Health and Rehabilitation, Zigong, Sichuan 643000, China; ^2^Department of Stomatology, Zigong Third People's Hospital, Zigong, Sichuan 643000, China

## Abstract

**Purpose:**

To perform a systematic review on the application of leukocyte- and platelet-rich plasma (L-PRP) in tendon models by reviewing *in vivo/in vitro* studies.

**Methods:**

The searches were performed via electronic databases including PubMed, Embase, and Cochrane Library up to September 2022 using the following keywords: ((tenocytes OR tendon OR tendinitis OR tendinosis OR tendinopathy OR tendon injury) AND (platelet-rich plasma OR PRP OR autologous conditioned plasma OR leukocyte- and platelet-rich plasma OR L-PRP OR leukocyte-richplatelet-rich plasma Lr-PRP)). Only *in vitro* and *in vivo* studies that assessed the potential effects of L-PRP on tendons and/or tenocytes are included in this study. Description of PRP, study design and methods, outcomes measured, and results are extracted from the data.

**Results:**

A total of 17 studies (8 *in vitro* studies and 9 *in vivo* studies) are included. Thirteen studies (76%) reported leukocyte concentrations of L-PRP. Four studies (24%) reported the commercial kits. *In in vitro* studies, L-PRP demonstrated increased cell proliferation, cell migration, collagen synthesis, accelerated inflammation, and catabolic response in the short term. In addition, most *in vivo* studies indicated increased collagen type I content. According to *in vivo* studies reporting data, L-PRP reduced inflammation response in 71.0% of studies, while it enhanced the histological quality of tendons in 67.0% of studies. All 3 studies reporting data found increased biomechanical properties with L-PRP treatment.

**Conclusions:**

Most evidence indicates that L-PRP has some potential effects on tendon healing compared to control. However, it appears that L-PRP works depending on the biological status of the damaged tendon. At an early stage, L-PRP may accelerate tendon healing, but at a later stage, it could be detrimental.

## 1. Introduction

Tendons, tight connective tissues, connect muscles and bones and transmit forces from the muscles to the bones, allowing the joint to move [1, 2]. Therefore, the tendon bears heavy mechanical loads and is prone to injury, which can affect tendon function [3]. Tendon diseases are common clinical diseases, mostly in athletes and inactive people, which constitute about approximately 30–50% percent of all sports injuries [4].

Nowadays, platelet-rich plasma (PRP) has gained a lot of interest in the treatment of tendon injuries [5]. PRP is a platelet concentrate obtained from whole blood through centrifugation [6]. A large body of literature suggests that PRP may have multiple potentials for tendon repair and regeneration because they store and release extensive growth factors, such as transforming growth factor-β (TGF-β), platelet-derived growth factor (PDGF), vascular endothelial growth factor (VEGF), and basic fibroblast growth factor (bFGF), insulin-like growth factor (IGF) [7, 8]. These growth factors are secreted by the dense granules, α-granules, and lysosomes in platelets [7, 8]. In addition to growth factors, other components including plasma, leukocytes, and residual erythrocytes also contain and/or release quite a few bioactive factors [7].

Although several studies have reported favorable therapeutic outcomes with PRP, some studies have shown less favorable results. These conflicting results are mainly ascribed to the different PRP preparation methods. A recent study investigated the cellular components of PRP and found significant differences in leukocyte concentrations between PRP preparations contrasted to platelets and fibrinogen [9]. According to whether they had more or fewer leukocytes than autologous blood, PRP can be divided into leukocyte- and PRP (L-PRP) and pure PRP (P-PRP) [10–14]. Moreover, the concentration and components of leukocytes have a significant effect on the function of PRP [15]. Most of the past research has not identified the specific components of PRP; however, because of the controversy over the therapeutic effect of PRP, a growing number of studies have identified the type of PRP based on whether it contains leukocytes or not [10, 13, 14, 16].

A systematic review will be conducted to determine if the delivery of L-PRP has promoted tendon healing in terms of methodology and reporting of the outcome. The mechanism of action of L-PRP and its efficacy in treating tendon injury will be studied in more detail and will ultimately demonstrate some benefits of L-PRP for tendon disorders *in vitro* and *in vivo*.

## 2. Method

### 2.1. Literature Search

We performed this literature review of *in vivo/in vitro* studies through foreign databases including PubMed, Embase, and Cochrane Library up to September 2022. The search terms were a combination of medical subject heading (MeSH) terms and their synonyms. The search query used was as follows: ((tenocytes OR tendon OR tendinosis OR tendinitis OR tendinopathy OR tendon injury) AND (platelet-rich plasma OR PRP OR autologous conditioned plasma OR leukocyte- and platelet-rich plasma OR L-PRP OR leukocyte-richplatelet-rich plasma OR Lr-PRP)).

### 2.2. Exclusion and Inclusion Criteria

Only *in vitro* and *in vivo* studies that assessed the potential effects of L-PRP on tendons and/or tenocytes are included in our review. We carefully reviewed the specific methodology of each included study, especially to accurately determine leukocyte concentration in the final PRP product used for tendon injection. L-PRP was characterized as PRP with a leukocyte concentration exceeding that of whole blood, whereas P-PRP was defined as PRP with a lower leukocyte concentration than that of whole blood [10–14]. When insufficient information was provided in the article, the study authors were contacted to acquire leukocyte concentrations. If the study authors did not record leukocyte concentrations, the manufacture's documentation of the PRP system they used was consulted to extract details about it. At the same time, according to the relevant literature analysis of PRP systems, it was decided which PRP system can produce L-PRP and included in this study, such as the Mini GPS III system, Smart PreP autologous platelet concentrate system, and platelet concentration collection system (PCCS), whereas the Arthrex autologous conditioned plasma double-syringe system, the Selphyl system, and the Endoret systems are known to produce leukocyte-poor PRP [15, 17–21]. With these methods, all formulations can be clearly classified into either class L or class P. Articles of undefined types of PRP and only P-PRP were excluded.

Of the studies that included additional therapeutic variables, only those trials that compared L-PRP directly to the control group (no treatment, saline solution, or control cell medium) were evaluated.

The study excluded randomized controlled trials and case studies. Only English-language studies published in peer-reviewed journals were considered.

**Figure 1:**
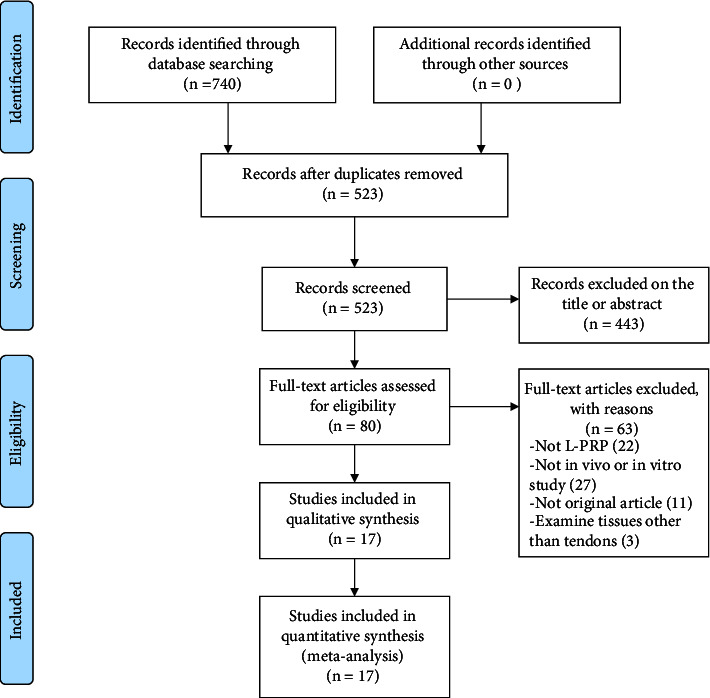
Preferred Reporting Items for Systematic Reviews and Meta-Analyses (PRISMA) diagram representing the process of individual study inclusion after application of the study algorithm and each of the exclusion criteria.

Two authors (YLL and MRY) searched separately, determined articles based on inclusion and exclusion criteria, and completed the PRISMA guidelines (Figure 1). Studies that met the criteria were cross-checked and included. In case of discrepancies, discussions and decisions were made by the senior researcher.

### 2.3. Data Extraction

Two authors extracted the article data and developed a standardized data table. Data collected included descriptions of PRP, study design and methods, outcomes measured, and results.

*In vitro* studies were analyzed for cell proliferation, cell migration, cell differentiation, the content of collagen types I and III, inflammatory mediation, and catabolic response. *In vivo* studies were analyzed for signal intensity in magnetic resonance imaging (MRI), collagen fibril diameters, histologic assessment of tendon repair, angiogenesis, inflammatory mediation, the content of collagen types I and III, cross-sectional area (CSA), lesion percent of the involved tendon, and biomechanical testing.

## 3. Results

### 3.1. Literature Search

In total, 740 articles were found through electronic searches; after removing 217 replicates, the remaining 523 related articles were analyzed and their titles and abstracts were screened, 443 articles were excluded due to the lack of adequate coverage of topics of interest. After full-text assessment, 63 additional articles were excluded because they did not meet the inclusion criteria (Figure 1). Thus, in total, 17 articles are included in this study. Of these, 8 are strictly *in vitro* studies [22–29] (Table 1), and 9 are strictly *in vivo* studies [30–38] (Table 2).

All included studies described the method of preparing PRP. Among them, thirteen studies (76%) recorded basic cytologic results involving white blood cells (WBC) counts of L-PRP [23–25, 27–31, 34–38]. Four studies (24%) were judged to be L-PRP relying on the commercial kits mentioned above [22, 26, 32, 33].

### 3.2. *In vitro* Studies

A total of 8 *in vitro* studies were conducted, of which 2 examined the effect of L-PRP on tendon stem cells (TSCs) (all two come from rabbits [28, 29]), 3 examined the effect on tendon explants (2 horses [24, 26], 1 human [22]), 1 examined the effect on tendon cell from rats [27], 1 examined the effect on human tendinopathic cells [25], and 1 examined the effect on human tenocytes [23] (Table 1).

Five of the studies stated the effect of L-PRP on cell proliferation, of which 4 studies showed significant increases in TSCs, tendinopathic cells, tenocytes, and tendon cells [23, 25, 27, 29]. In addition, 1 of them demonstrated a significant decrease in TSCs [28] (Table 3).

Only 1 *in vitro* study reported the influence on cell migration, which illustrated increased cell migration after the application of L-PRP [25].

Less is known about the influence of L-PRP on cell differentiation. Among the eight *in vitro* studies, 2 reported cell differentiation data, with 1 study demonstrating significant increases in the differentiation of TSCs into active tenocytes [29] and another demonstrating nontenocyte differentiation of TSCs [28].

The effect of L-PRP on collagen types I and III was reported in 7 studies, 6 of which showed significant increases in collagen I and III or collagen I/collagen III ratio [22–24, 26, 28, 29], and only 1 showed a significant decrease [25].

Five of these studies reported the influence of L-PRP on inflammatory mediation, and all 5 studies showed a significant increase [22, 24, 25, 28, 29].

Finally, the catabolic response was also analyzed. 4 of 5 studies reported L-PRP to have significant increases in it [22, 24, 25, 29], and the remaining 1 study demonstrated no difference [26].

### 3.3. *In vivo* Studies

In the 9 *in vivo* studies, the animal models used included 5 rabbits [31, 33, 34, 36, 38], 2 mice [35, 37], 1 rat [32], and 1 horse [30]. Animal models were established by different methods, of which 4 were injured by surgery [30, 32, 35, 37], 3 were established by collagenase injection [34, 36, 38], and 2 were normal tendons [31, 33] (Table 2).

Signal intensity in MRI with L-PRP treatment is analyzed in 3 studies, 2 of the studies demonstrated a significant decrease in T2 mapping signal intensity in MRI [34, 36], while the third study showed no change [38] (Table 4).

Three studies reported data on collagen fibril diameters, and all of them demonstrated significant increases with the use of L-PRP [34, 36, 38].

Six of 9 *in vivo* studies reported histologic assessment of tendon repair after L-PRP treatment. Four of the studies reported that L-PRP significantly improved the quality of tendon injury tissue [34–37], whereas 2 studies demonstrated no change [32, 38]. Three studies reported data on angiogenesis, with 2 of the studies reporting L-PRP significantly accelerated angiogenesis of tendon, which then gradually decreases with the tendon healing process [35, 37], whereas the third study showed a significant decrease [34].

The effect of inflammatory mediation with L-PRP treatment was most widely researched. Seven of the 9 *in vivo* studies reported inflammatory mediation. Among them, 5 studies showed a significant decrease [31, 33, 34, 36, 37] (4 of them reported an initial increase, but a decrease over time), and 2 studies showed no difference [32, 38].

The effect of L-PRP on collagen types I and III was also analyzed in a small amount of literature. Three studies reported collagen type I, 2 of which showed a significant increase [34, 36] whereas the third study showed no difference [38]. Meanwhile, 2 studies reported collagen type III, and all of 2 showed a significant decrease [34, 36].

Three studies reported data on the cross-sectional area (CSA) or lesion percent of the involved tendon. Three of them reported CSA, 2 of which showed a significant decrease [34, 36], and 1 showed no difference [38]. Two studies reported lesion percent of the involved tendon, 1 showed a significant decrease [36], and 1 showed no difference [38].

For biomechanical testing, such as failure load, tensile stress, and stiffness, all three studies showed that L-PRP significantly improved biomechanical properties [30, 32, 34].

## 4. Discussion

Nowadays, the application of PRP has progressed rapidly without a large amount of data to support its safety or clinical efficacy [39]. Although many meta-analyses have reported the clinical role of PRP, the results are still confusing, somewhat favorable, somewhat unhelpful, and somewhat even harmful [13, 14, 40, 41]. Literature on PRP preparation methods as well as platelet concentration and cytology reports are inconsistent. Among them, leukocyte concentration is one of the most vital factors influencing PRP function [13, 15, 24, 40].

In this literature review, we searched basic science articles on the use of L-PRP on tendon disease. Unfortunately, because the number of studies included is too small, data could only be qualitatively analyzed. Further research must be conducted to support our findings in the future.

In *in vitro* studies, parameters such as cell proliferation, the content of collagen types I and III, catabolic response and inflammatory mediation have generally been used to assess the efficacy of L-PRP treatment for tendon repair, while in *in vivo* studies, the criteria of evaluation of L-PRP treatment are histologic assessment and inflammatory mediation. Most evidence indicates that L-PRP has several beneficial effects on these parameters compared to control.

Four of 5 *in vitro* studies reporting cell proliferation showed a significant increase in the proliferative ability of tendon associated cells by L-PRP, and merely one showed a significant decrease. Tendons are rich in collagen, with the most abundant collagen being type I collagen, which accounts for approximately 95% of total collagen. Six of 7 *in vitro* studies reported collagen type I and III significant increase, and only one showed significant decrease. Meanwhile, in the *in vivo* studies, 2 of 3 studies that reported collagen type I demonstrated significant increases in the content of collagen type I, with the remaining 1 study showing no difference. Both 2 *in vivo* studies reported that L-PRP significantly decreases the content of collagen types III. After tendon injury, collagen type I content is down-regulated and the synthesis of type III collagen is enhanced. As the tendon heals, type III collagen gradually converts into collagen type I. Thus, it seems that L-PRP could promote collagen type I synthesis but not collagen type III to facilitate tendon repair in both *in vivo* and *in vitro* studies.

Furthermore, all of 3 *in vivo* studies that reported collagen fibril diameters demonstrated that L-PRP significantly increases collagen fibril diameters. In *in vivo* studies, 4 of the 6 studies that reported the histological changes of tendons showed a significant increase in the quality of tendon healing by L-PRP, and the other 2 studies reported no difference. Parameters used to assess the quality of tendon histological repair include better fiber structure and arrangement, less cell density, less angiogenesis, and less inflammation.

Four of the 5 *in vitro* studies reported that L-PRP significantly increases catabolic cytokines, such as matrix metalloproteinases-9/11 (MMP-1/9). MMPs are zinc endopeptidases that regulate the extracellular matrix components, which may inhibit matrix formation. Some literature have proposed that L-PRP could have both catabolic and anabolic properties [31, 42]. Nevertheless, this function appears to be time-dependent as there is less benefit in delaying L-PRP administration after the early injury.

All the 5 *in vitro* studies reporting inflammatory mediation revealed that L-PRP significantly exacerbates inflammation in a short time. However, 5 of 7 *in vivo* studies demonstrated that L-PRP significantly decreases inflammation, 4 of which showed a short term increase but a long-term decrease while the other 2 studies showed no difference. Consistent with inflammation mediation, 2 of 3 *in vivo* studies reported that L-PRP significantly decrease signal intensity in MRI in T2 mapping, which means a decrease in local inflammation. It is worth noting that in these two works of literature, animal tendon injury models were established by injection of collagenase, and L-PRP treatment was applied 1 week after collagenase injection, which is generally considered to be the acute stage of tendon injury or the early stage of tendinopathy. However, when L-PRP treatment was applied 4 weeks after collagenase injection (usually considered to be later stageof tendon injury), there were no significant inflammation changes observed by both MRI T2 mapping and inflammatory mediation in histology. Interestingly, this is consistent with the histologic assessment of tendon repair. The application of L-PRP to the early phase of collagenase injection improved the histology results, while there was no difference when applied to the later phase of collagenase injection. It demonstrates that the effects of L-PRP are multifactorial, and the timing of application may alter its anti-inflammatory capacity and overall efficacy.

Inflammation is an essential process for tendon healing, and leukocytes contained in L-PRP are instrumental. During the inflammatory phase, inflammatory cells such as neutrophils, monocytes, and macrophages migrate to the damaged tissue and remove necrotic material by phagocytosis [43–45]. Several studies have shown that early administration of L-PRP accelerates the repair of tendinopathy in rabbits more than late administration, suggesting that L-PRP may promote inflammation in the early stage to accelerate tendon healing, while the beneficial effect of L-PRP in the late stage of tendon lesion was not obvious [29, 34, 36, 38].

Finally, for tendon function, all 3 *in vivo* studies demonstrated improved biomechanical testing, such as the failure load, stiffness, and ultimate tendon stress, meaning that L-PRP improves tendon outcomes and allows the tendon to return to life and exercise earlier.

## 5. Limitations

In this study, the lack of uniform methodology and reporting of results was the major limitation for more detailed and in-depth analysis and comparison, as previously described. This study also lacked a risk of bias assessment for the included studies. However, there is currently no effective tool to assess the existence of bias in basic scientific research. A tool that could provide a more objective assessment of our topic should be available to evaluate the quality and bias risks of these basic scientific studies.

*In vivo* and *in vitro* studies also have some inherent limitations. For *in vivo* studies, tendon injuries in animals are distinctly different from those in humans. In animals, the lesions are usually small, and the tendon thickness is thinner. In addition, animal models with surgical incisions or injections of collagenase cannot truly mimic clinical human tendon disease.

Because of these limitations, it is hard to apply our findings to the clinic. However, basic scientific research remains essential to evaluate the efficacy and mechanisms of L-PRP for tendon therapy.

## 6. Conclusions

In this review of the literature, it was found that L-PRP is beneficial to these parameters in comparison with controls, including angiogenesis, collagen synthesis, inflammation, and biomechanical property. It appears that L-PRP works depending on the biological status of the damaged tendon. At an early stage, L-PRP may accelerate tendon healing, but at a later stage, it could be detrimental. Furthermore, study methodology, including the timing of PRP administration, is not standardized across studies, which hinders comparisons of the efficacy of L-PRP.

For a better understanding of L-PRP's role in tendon pathology, more rigorous controlled experiments and consistent evaluation criteria must be set up in future basic scientific and clinical studies.

## Figures and Tables

**Table 1:** *In vitro* studies on L-PRP for tendon models.

Study (author, Year)	System used to Obtain L-PRP/cytologic findings	Type of cells	Study design	Time of analysis	Outcomes measured	Results (L-PRP compared with control)
Zhang et al. [28]	Leukocytes in L-PRP were 4 times higher than whole blood; leukocytes in P-PRP were 2 times lower than whole blood	TSCs	L-PRP was prepared from rabbits. TSCs were isolated from the patellar tendons of healthy rabbits. TSCs cultured and treated with DMEM with FBS (control group), P-PRP, or L-PRP	1 week	Cell proliferation, collagen production, apoptosis, expression of specific growth factors, nontenocyte-related genes, and inflammation-related genes	Cell proliferation↓, collagen production↑, apoptosis↑, expression of specific growth factors (VEGF, EGF, TGF-*β*1, and PDGF)↑, nontenocyte-related genes (PPAR, SOX-9, and Runx-2)↑, inflammation-related genes (IL-1*β* and mPGES)↑

Yu et al. [27]	5.404 ± 0.646 × 10^3^/ml (WBC in whole blood 6.568 ± 1.029 × 10^3^/ml (WBC in L-PRP)	Tendon cell	L-PRP was prepared from rats. Tendon cells were obtained from rats, were cultured, and sampled from normal fibroblast shape. Cells were then cultured in serum-free medium under five different conditions: Medium only (control), or 0.1%, 0.5%, 1% or 2% L-PRP	24 h	Cell proliferation, expression of specific growth factor	Cell proliferation↑in a dose-dependent manner, expression of specific growth factors (TGF-*β*1 and PDGF) ↑

Schnabel et al. [26]	SmartPReP2 system (Harvest Technologies, Plymouth, MA)	Tendon explants	L-PRP was prepared from young adult horses. Tendon explants from horse normal forelimb flexor digitorum superficialis tendon cultured and treated with whole blood, 10% plasma (as control), L-PRP, PPP, or bone marrow aspirate (BMA) at concentrations of 100%, 50%, or 10%	3 days	Expression of specific growth factor, gene expression of the matrix molecules and catabolic molecules	Expression of specific growth factor (TGF-*β*1 and PDGF-BB)↑, gene expression of the matrix molecules (COL1, COL3 and COMP) ↑, no increase in catabolic molecules (MMP-3 and MMP-13)

Zhou et al. [29]	5.404 ± 0.646 × 10^3^/ml (WBC in whole blood); 6.568 ± 1.029 × 10^3^/ml (WBC in L-PRP)	TSCs	L-PRP and P-PRP were prepared from rabbits. TSCs were isolated from the patellar tendons of rabbits. TSCs were cultured and treated with control (DMEM + 2%FBS), L-PRP, or P-PRP	2 weeks	Cell proliferation, differentiation of TSCs into active tenocytes, catabolic marker genes, inflammatory genes, tenocyte-related proteins.	Cell proliferation↑in a dose-dependent manner, differentiation of TSCs into active tenocytes↑, catabolic marker genes (MMP-1, MMP-13)↑, inflammatory genes (IL-1*β*, IL-6, TNF-*α*)) ↑, tenocyte-related proteins (COL1 and COL3)↑

Rubio-Azpeitia et al. [25]	L-PRP is called by authors 4.7 ± 3.8 × 10^3^/*μ*L (leukocytes in L-PRP).	Tendinopathic cells	L-PRP, PPP, and P-PRP were prepared from 4 healthy donors (2 men and 2 women). Tendon cells were isolated from chronic tendinopathy tissue which comes from supraspinatus tendon biopsy samples obtained from patients undergoing arthroscopic shoulder surgery and cultured and treated with PPP (as control), P-PRP, or L-PRP	96 hours	Cell migration, cell proliferation, expression of genes associated with matrix turnover, inflammatory proteins	Cell migration↑, cell proliferation↑, expression of genes associated with matrix turnover (COL1 and COL3↓, MMP-1 expression↑, Decorin, fibronectin, and aggrecan↓), inflammatory proteins (IL-6↑)

Cross et al. [22]	L-PRP: Biomet GPS III Mini platelet concentrate kit; P-PRP: Arthrex autologous conditioned plasma double Syringe system	Tendon explants	Venous blood was collected from a healthy human volunteer population. Tendon explants were taken from the lateral aspect of patients' chronically torn supraspinatus tendons. Tendons were separated into group 1 (moderate tendinopathy) and group 2 (severe tendinopathy) according to prepared and scored tendon samples histologically. Tendons were cultured and treated with P-PRP, L-PRP, or control media (DMEM with FBS)	96 hours	Matrix gene expression, catabolic gene expression	Matrix gene expression (COL1: COL3 ratio) ↑ (group 1), catabolic gene expression (MMP-9↑ (group 2), IL-1*β* (group 2))

McCarrel et al. [24]	(5 to10) × 10^3^ (WBC in standard PRP, made by SmartPReP2 system); (25 to 30) × 10^3^ (WBC in L-PRP); (0 to 2) × 10^3^ (WBC in P-PRP)	Tendon explants	PRP was prepared from young adult horses. Tendon explants from horse normal forelimb flexor digitorum superficialis tendon cultured and treated with standard PRP, L-PRP, P-PRP or control (10% plasma in DMEM)	72 h	Growth factor, matrix gene expression, catabolic gene expression	Growth factor (PDGF↑), matrix gene expression (COL1: COL3 ratio↑, COMP↑), catabolic gene expression (MMP-13↓, IL-1*β*↑, TNF-*α*↑)

Lin et al. [23]	L-PRP is called by authors; 4.7 ± 3.8 × 10^3^/*μ*L (leukocytes in L-PRP)	Tenocytes	PRP was collected from 3 patients during rotator cuff repair. Tenocytes were isolated from torn human supraspinatus tendons during arthroscopic rotator cuff repair of 3 patients with moderate degenerative rotator cuff tears and were cultured and treated with control (only culture medium supplementation), P-PRP, or L-PRP	3 days	Gene analysis, tenocyte proliferation	Gene analysis (TNC, COL1, COL3, and SCX) ↑, tenocyte proliferation↑

↑, significant increase; ↓, significant decrease; n. a., not affected; TSCs, tendon stem cells; L-PRP, leukocyte- and platelet-rich plasma; P-PRP, pure platelet-rich plasma; PPP, platelet poor plasma; DMEM, Dulbecco's modified Eagle's medium; FBS, fetal bovine serum; VEGF, vascular endothelial growth factor; EGF, epidermal growth factor; TGF-*β*1, transforming growth factor–*β*1; PDGF, platelet-derived growth factor; PPAR, peroxisome proliferator-activated receptor; SOX-9, SRY (sex determining region Y)–box 9; Runx-2, runt-related transcription factor 2; IL-1*β*/6, Interleukin-1*β*/6; COMP, cartilage oligomeric matrix protein; mPGES, membrane-associated prostaglandin synthase; WBC, white blood cell; COL1, type I collagen; COL3, type III collagen; MMP, matrix metalloproteinase; TNF-*α*, tumor necrosis factor-*α*; TNC, tenascin-C; SCX, scleraxis.

**Table 2:** *In vivo* studies on L-PRP for tendon models.

Study (author, Year)	System used to Obtain L-PRP/cytologic findings	Type of animals	Model establish	Study design	Outcomes measured	Results (L-PRP compared with control)
Kobayashi et al. [35]	The leukocyte concentration of the L-PRP was approximately 2.5-fold higher than that of the whole blood, whereas the PPP contained very few leukocytes	Mice	Full-thickness defects were created in the central third of the patellar tendon of the right hindlimbs via microsurgery technique	The prepared allogenic L-PRP gel was applied on the defect of the patellar tendon (L-PRP group), or untreated control group	Mice were sacrificed at 2, 4, 6, 8, and 10 weeks after the operation, with histological sections obtained in each time point. Semi-quantitative histological evaluation was performed in accordance with the Bonar score. The ratio of the collagen fibers to the entire tendon tissue (FT ratio) was measured as a quantitative histological evaluation	The total Bonar score in the L-PRP group was significantly lower than in control group. The vascularity score was significantly higher in the L-PRP group at 2 and 4 weeks, while the collagen arrangement score was significantly lower in the L-PRP group at 8 weeks. Based on a quantitative evaluation, the recovery speed of the patellar tendon determined by FT ratio was significantly faster in the PRP group than in the control group at 6 and 8 weeks

Li et al. [36]	25.04 ± 3.96 × 10^3^/*μ*l (WBCs in L-PRP-1); 6.93 ± 1.72 × 10^3^/*μ*l (WBCs in whole blood 1); 22 ± 5.04 × 10^3^/*μ*l (WBCs in L-PRP-2) 6.03 ± 1.26 × 10^3^/*μ*l (WBCs in whole blood 2).	Rabbit	Local collagenase injection on the rabbit achilles tendon	After collagenase induction, rabbit achilles tendons were randomly treated with an injection of 0.2 mL of either L-PRP or saline at 1 week (PRP-1 group) and 4 weeks (PRP-2 group), represently	Six weeks after collagenase induction, outcomes were assessed by MRI, catabolic cytokines, gene expression, histology, TEM	PRP-1: MRI (cross-sectional area and lesion percent↓, signal intensity ↓ in T2 mapping), catabolic cytokines (IL-10↑, IL-6↓), gene expression (COL1 ↑), histology (general scores↑), TEM (collagen diameter↑, mean collagen fibril diameter↑, lower fibril density↓, volume fraction↑); In PRP-2, MRI (cross-sectional area↑), gene expression(Col 3↑, MMP-1↑, and MMP-3↑), histology (n. a.), TEM (collagen diameter↓, mean collagen fibril diameter↓, lower fibril density↑, volume fraction↓)

Dragoo et al. [31]	21.52 ± 9.4 × 10^3^/*μ*L (mean WBC count for L-PRP); 2.61 ± 0.78 × 10^3^/*μ*L (mean WBC count for whole blood); 0.63036 ± 0.475 × 10^3^/*μ*L (mean WBC count for P-PRP)	Rabbit	Healthy patellar tendons	In the control animals, one patellar tendon was injected with 2 mL autologous whole blood, and the other was injected with 2 mL sterile saline. In the experimental animals, one patellar tendon was injected with 2 mL L-PRP, and the other was injected with 2 mL P-PRP.	Animals were euthanized at 5 or 14 days after injection. Tendons were harvested and stained using hematoxylin and eosin and scored semi-quantitatively for total WBCs, mononuclear cells (macrophages and lymphocytes), polymorphonuclear cells (PMNs), vascularity, fiber structure, and fibrosis	At 5 days after injection, tendons treated with L-PRP had significantly greater vascularity, and fibrosis and overall tendon scores (compared with whole blood). tendons treated with L-PRP had significantly greater mean scores for fiber structure disruption, mononuclear cells, vascularity, and fibrosis and total tendon scores (compared with saline); There were no significant differences at 14 days

Harris et al. [33]	PRP was prepared with platelet concentrate Collection system [PCCS]	Rabbit	Normal achilles tendon, medial collateral ligament	Rabbits were injected with 0.5 mL. L-PRP in the right or left achilles tendon, medial collateral ligament. Saline solution was injected on the contralateral side as a control	The soft tissues were examined histologically at two weeks and six weeks, and soft tissues from rabbits that had been reinjected at six weeks were examined at twelve weeks	Inflammatory skin lesions were visible at forty-eight hours at superficial platelet-rich plasma sites. All lesions resolved by six days. Tendon and ligament sites showed new collagen deposition. Histological examination of platelet-rich plasma injection sites at six and twelve weeks demonstrated a persistent but diminished inflammatory infiltrate

Ersen et al. [32]	L-PRP was prepared with the GPS 3 MINI SYSTEM (Biomet Biologics)	Rat	The supraspinatus tendons of rats were detached from their insertion on the humerus	The rats were divided into 4 groups: An untreated control group, primary-repair-only group, repair plus 0.3 mL injected PRP group into the tendon–bone interface, repair plus 0.3 mL absorbed PRP group from a sponge.	All rats were killed at 8-week after surgery, and the supraspinatus muscle was removed from the supraspinatus fossa leaving the tendon–bone junction intact. The supraspinatus tendon was evaluated biomechanically and histologically at week 8	Cuffs repaired with L-PRP had significantly greater mean (SD) load-to-failure rates and stiffness than did cuffs repaired without L-PRP. The collagen fiber orientation in L-PRP-treated groups was more mature oriented and aligned

Bosch et al. [30]	The leukocyte concentration of the L-PRP was 6-fold higher than that of the serum	Horses	Tendon lesions were created surgically in the superficial digital flexor tendons (SDFT) of both front limbs of horses	One of horse front limbs was treated with L-PRP and the other with saline under ultrasonographic guidance	After 24 weeks, the tendons were harvested for biochemical, biomechanical, and histological evaluations	Collagen, glycosaminoglycan, and cellularity was higher in L-PRP-treated tendons. The repair tissue in the L-PRP group showed a higher strength at failure and Elastic Modulus. Histologically, L-PRP-treated tendons featured better organization of the collagen network and signs of increased metabolic activity

Yan et al. [38]	Relative to whole blood, L-PRP had a 3.7-fold enrichment of WBCs	Rabbit	Local collagenase injection on the rabbit achilles tendon	4 weeks after collagenase induction, animals were randomly assigned to 3 group to treat with 0.2 mL of P-PRP, L-PRP or saline	Healing outcomes were assessed at 4 weeks after therapy MRI, cytokine quantification, RT-PCR analysis of gene expression, histology, and TEM	L-PRP: MRI (n. a. in cross-sectional area, lesion percent of the involved tendon and signal intensities in T2 mapping), histological score (n. a.), catabolic cytokine IL-6, IL-1*β* and TNF-*α* by ELISA (n. a.), expression of COL1 (n. a.), TEM (fibril density↓, collagen fibril diameters↑, mean fibril area↑, volume fraction↑), MMP–1 and MMP-3↓

Jiang et al. [34]	The WBCs of L-PRP was 3.0-fold compared with whole blood	Rabbit	Local collagenase injection on the rabbit achilles tendon	1 week later after collagenase induction, treatments were applied randomly on local achilles tendon lesions with 0.2 mL of L-PRP, P-PRP or saline	At 3 and 6 weeks after the collagenase injection, outcomes were evaluated by histology, MRI, gene expression analysis, immunohistochemistry, TEM and biomechanical testing	L-PRP group: MRI (lower T2 signal intensity, smaller diameter and cross-sectional area). Histology (better scores, better fiber structure, less angiogenesis, less inflammation), gene expression (COL1↑, COL3↓ and CD163↑). TEM (lower fibril density, longer mean fiber diameter, largest fibril area, higher volume fraction), biomechanical testing (failure load↑, tensile stress↑, stiffness↑)

Nishio et al. [37]	L-PRP had abundant populations of granulocytes (65.8 × 10^3^ ± 18.5^*∗*^10^3^/*μ*L) and lymphocytes (106.6 × 10^3^ ± 21.0 × 10^3^/*μ*L) L-PRP versus whole blood (*p* < 0.0001)	Mice	Full-thickness defects were created in the central third of the patellar tendon using a microsurgery technique	Mice were treated on the patellar tendon defect either with L-PRP, L-PRP, or without PRP (control group)	On postoperative day 7, 14, 28, or 42 days, histological analyses were performed to evaluate the tendon healing using Bonar score as semiquantitative histological scoring system. Flow cytometric analyses were performed to count the number of GFP-positive cells around repaired patellar tendon	L-PRP: Better histology score, flow cytometric analyses (number of GFP-positive MP↑), the number of pro-inflammatory MPs (M1) ↑ on day 4 and 7, the number of anti-inflammatory MPs (M2) ↑ on day 7

↑, significant increase; ↓, significant decrease; n. a., not affected; L-PRP, leukocyte- and platelet-rich plasma; P-PRP, pure platelet-rich plasma; PPP, platelet poor plasma; MRI, magnetic resonance imaging; TEM, transmission electron microscopy; RT-PCR, real-time polymerase chain reaction; COL1, type I collagen; COL3, type III collagen; MP, macrophages; IL-1*β*/6/10, Interleukin-1*β*/6/10; MMP, matrix metalloproteinase; TNF-*α*, tumor necrosis factor-*α*; ELISA, enzyme-linked immunosorbent assay.

**Table 3:** Variables reported *in vitro* (L-PRP compared with control).

Outcome	Studies reporting, *n* (%)	Significant increase, *n*	No significant change, *n*	Significant decrease, *n*
Cell proliferation	5 (62.5%)	4		1
Cell migration	1 (12.5%)	1		
Cell differentiation	2 (25%)	1		1
Collagen types I and III	7 (87.5%)	6		1
Inflammatory mediation	5 (62.5%)	5		
Catabolic response	5 (62.5%)	4	1	

L-PRP, leukocyte- and platelet-rich plasma.

**Table 4:** Variables reported *in vivo* (L-PRP compared with control).

Outcome	Studies reporting, *n* (%)	Significant increase, *n*	No significant change, *n*	Significant decrease, *n*
Signal intensity in MRI (T2 mapping)	3 (33%)		1	2 (1 study showed decrease on L-PRP application of early stage while no change on application of later stage)
Collagen fibril diameters	3 (33%)	3 (1 study showed increase on L-PRP application of early stage while it decrease on application of later stage)		
Histologic assessment of tendon repair	6 (66%)	4 (1 study showed short term decrease, but long term increase; 1 study showed increase on L-PRP application of early stage while it showed no change on application of later stage)	2	
Angiogenesis	3 (33%)	2		1
Inflammatory mediation	7 (77%)		2	5 (4 study showed short term increase, but long term decrease; 1 study showed decrease on L-PRP application of early stage while it showed no change on application of later stage)
Collagen types I	3 (33%)	2 (1 study showed increase on L-PRP application of early stage while it showed no change on application of later stage)	1	
Collagen types III	2 (22%)			2 (1 study showed decrease on L-PRP application of early stage while it increase on application of later stage)
Cross-sectional area (CSA)	3 (33%)		1	2 (1 study showed decrease on L-PRP application of early stage while it increase on application of later stage)
Lesion percent of the involved tendon	2 (22%)		1	1 (1 study showed decrease on L-PRP application of early stage while it showed no change on application of later stage)
Biomechanical testing	3 (33%)	3		

L-PRP, leukocyte- and platelet-rich plasma; MRI, magnetic resonance imaging.

## Data Availability

All data generated or analyzed during this study are included in this article.
